# An Account of the Quassia Polygama, or Bitter-Wood of Jamaica; and of the Cinchona Brachycarpa, a New Species of Jesuit's Bark Found in the Same Island

**Published:** 1794

**Authors:** John Lindsay

**Affiliations:** Surgeon in Westmoreland, Jamaica.


XIV. *An Account of the Quaffia Polygama, or Bitter-wood of Jamaica; and of the Cinchona Brachycarpa, a new Species of Jefuit's Bark found in the fame Ifland. By Mr.* John Lindfay, *Surgeon in Weftmoreland, Jamaica.* Vide *Tranfactions of the Royal Society of Edinburgh,* Vol. III.

THE tree, which is the fubject of this paper, has been long known in Jamaica, and in fome other iflands in the Weft Indies, not only as an excellent timber, but as affording an ufeful medicine in putrid fevers and fluxes. In Jamaica it is called *Bitter wood*; in the windward iflands, *Bitter afh*; and in the French iflands it is known by the name of *Excorfier.*

The

The bark has for fome time been prefcribed by medical practitioners in Jamaica, and confiderable quantities of it have been exported to England for the purpofes of the brewers of ale and porter. On thefe accounts Mr. Lindfay has been induced to communicate the prefent defcription of this tree to the public.

Prefixed to this defcription is a fhort hiftorical account of the tree in queftion, collected from preceding writers.

The firft of thefe is Sir Hans Sloane, who, in his vifit to Barbadoes, having noticed the Bitter wood, has given the following defcription of it in his Catalogue; "Melanomma et melanoxylum, "arbor laurifolia nucifera, gemmis nigricanti-"bus, Americana:" and refers to Plukenet's Phytographia, Tab. 205, fig. 3; but the plant there delineated, our author obferves, is different from the prefent.

Dr. Patrick Browne, and after him Mr. Long, in their Hiftories of Jamaica, mention this tree by the names of *Xylopicrum*, *Xylopia glabra*, *Bitter-wood*, or *Bitter Afh*. Mr. Long, in fpeaking of the *Quaffia Amara*, thinks the Bitter Afh of St. Chriftopher's is the fame, but does not feem to know whether the Bitter Afh has been found in Jamaica.

Dr. William Wright, in his Account of the Medicinal Plants growing in Jamaica *, mentions this tree under the title of *Picrania Amara*, a new genus belonging to the clafs *Pentandria Monogynia*; and fays it is ufed in putrid fevers as an antifeptic, and that lefs of it will do than of the *Quaffia Amara* of Linnæus. Dr. Wright, our author obferves, was naturally led to place this tree in the clafs and order he has done, from finding hermaphrodite flowers and feeds on the fame tree; but at the fame time he was aware that this tree has a great affinity to the genus Quaffia.

The laft writer referred to by our author is Dr. Olof Swartz, concerning whom he remarks, that having examined moft of the plants in Jamaica, he probably had feen this tree in flower and fruit, as he ftyles it in his Prodromus, "*Quaffia Excelfa*, floribus herma-"phroditis 5dris paniculatis, foliis impari-pin-"natis, foliolis oppofitis petiolatis, petiolo "nudo."

Mr. Lindfay fuppofes that no other defcription of this tree has yet appeared; of courfe he had had no opportunity of knowing, at the

* London Medical Journal, Vol. VIII. p. 275.

time time his paper was written, that Dr. Swartz, in the fame year in which he publifhed his Prodromus, communicated to the Royal Academy of Sciences at Stockholm a botanical defcription and figure of the Quaffia Excelfa, which the Academy have publifhed in their Tranfactions for the year 1788.

We now come to Mr. Lindfay's Account of this tree, which he defcribes as being very common in moft of the woodlands in Jamaica; and as being beautiful, tall, and ftately. He has meafured one, it feems, which was an hundred feet in length, and ten feet in circumference.

He obferves that the trunk is ftraight, fmooth and tapering, fending off its branches towards the top; that the outfide bark is pretty fmooth, of a light gray or afh colour, from various lichens; that the bark of the roots is of a yellow caft, fomewhat like the Cortex Simarouba; and that the inner bark is tough, and compofed of fine flaxy fibres; that the wood is of a yellow colour, tough, but not very hard; that it takes a good polifh, and is ufed as flooring; that the leaves are fub-alternate; the fmall leaves being in pairs, from five to eight, ftanding oppofite to each other on fhort foot-ftalks, and and ending with an odd one; that they are of an oblong oval ſhape, and pointed; the ribs reddiſh, and the young leaves covered with a fine browniſh down; that the flowers come out in bunches or cluſters from the lower part of the laſt ſhoot before the leaves, and ſtand on round footſtalks; that the flowers are ſmall, of a yellowiſh green colour, with a very ſmall calyx; and that the male or barren tree produces flowers nearly ſimilar to the hermaphrodite, but which have only the rudiments of a ſtyle.

The fruit, he tells us, is a ſmooth black *drupa*, round ſhaped, and of the ſize of a pea. There is but little pulp, and the nut covers a round kernel. Theſe *drupæ*, he obſerves, are generally three, ſometimes two, and often only one, attached ſideways to a roundiſh fleſhy receptacle. It flowers in October and November, and its fruit is ripe in December and January.

Except the pulp of the fruit, every other part of this tree, we are told, has an intenſely bitter taſte. From this quality, Sir Joſeph Banks, Dr. Solander, and Dr. Wright, were induced, it ſeems, to give it the name of *Picrania Amara*. In taſte and virtues, our author has found it nearly equal to the Quaſſia of Surinam, rinam *, and has been credibly informed that it is ſold in London for the Quaſſia Amara, and he thinks, it may be ſafely uſed in all caſes where

* From a paper entitled " of the true Quaſſia Amara, " and of the falſe (*Om den rätte Quaſſia Amara, og om* " *den falſke*)" lately publiſhed by Mr. N. Tönder Lund, in the Tranſactions of the Natural Hiſtory Society at Copenhagen, it would ſeem that what has been generally imported into Europe under the name of Quaſſia Amara, is ſpurious. ' Mr. Von Rohr,' ſays the author of the paper in queſtion, ' who in the years 1783, 4, and 5, viſited different parts of the Continent of America, and ſeveral of the Weſt India iſlands, and collected many rare ' plants, has ſent to me, among other things, a ſpecimen ' of the Quaſſia Amara, and with it the following note: " In my whole voyage I ſaw only a ſingle wild ſhrub of " Quaſſia Amara, and that was near the river Tamaco, in " the neighbourhood of St. Martha. The planters in Surinam and Cayenne cultivate it on account of the magnificence of its flowers, and its uſe. In Surinam, the " flowers alone are uſed as tea. The wood is extremely " dear; and I can with truth ſay that I know not where I " could buy a ſingle pound of it. The ſtem never exceeds " two inches in diameter. Had I been deſirous of extirpating this ſhrub from the neighbourhood of the Tamaco, I might perhaps have got together ten pounds of " it. It is certain, therefore, that impoſtors have ſent " into Europe, under the name of Quaſſia Amara, the " wood of another ſpecies of Quaſſia, which has the apwhere that drug has been thought proper, whether as an antiseptic, or in cases of weakness in the stomach and bowels. It may either, he observes, be given alone, or joined with the Peruvian bark.

He has seen, he tells us, the happiest effects from the use of this medicine in obstinate remitting fevers from marsh miasmata, in agues which had resisted the use of Peruvian bark, and in dysenteries of long standing. In Jamaica, it seems, it is in daily use in dropsies from debility, either in simple infusion or tincture, or joined with aromatics and chalybeates.

Dr. Drummond, an eminent physician in Jamaica, is said to prescribe it with great suc-

"pearance of an ash tree, and which is likewise bitter. In "a word, you may be assured that any specimen of Quassia, the stem of which, including the bark, is more than "two inches in diameter, is not the true Quassia." From 'this account," adds Mr. Lund, "it seems pretty certain, that the pieces of wood which are met with in the 'shops, and which have more the appearance of timber 'than of a medicine, are not the true Quassia, but are 'procured from that species of it which the English call 'the bitter ash."—See *Skrivter af Naturhistorie-Selskabet.* 8vo. Copenhagen, 1790. Vol. I. Part 2, page 68. Editor.

cess cess in the above cases, as well as in amenorrhœa, chlorosis, dyspepsia, and in that species of pica, called *Dirt-eating*, so fatal to a number of negroes.

The bark of the tree, but especially the wood, Mr. Lindsay observes, is intensely bitter. He has used both in various forms.

The bark, it seems, is difficult to be reduced to powder. The dose, he observes, is from 15 grains to 1 drachm, either by itself, or joined with the Peruvian bark.

Mr. Lindsay has employed the wood as well as the bark of this tree, both in infusion and decoction; for the former he directs the proportions to be from two drachms to half an ounce of the bark or wood to a pint of water; and for the latter the same quantities to a pint and a half of water, which is to be boiled to a pint. Of either of these the dose is directed to be a wine-glass full every three, four, or six hours, according to circumstances.

In certain cases of dropsy, in amenorrhœa and chlorosis, he joins to this remedy aromatics and other medicines; and in worm fevers, the cabbage bark or other vegetable anthelmintics.

Mr. Lindsay gives the following botanical description of the Quaffia Polygama*. This description is accompanied with engraved figures

* As we think it will be a gratification to many of our readers to fee Dr. Swartz's defcription of the fame tree, under the name of Q. Excelfa, we fhall here infert it.

" *Arbor* excelfa.

" *Truncus* craffus. *Cortex* cinereus, rimofus.

" *Lignum* duriffimum, album.

" *Rami* patentes.

" *Folia* pinnata cum impari, alterna, fparfa. *Petioli* teretes glabri.

" *Foliola* petiolata, 4-6juga, oppofita, elliptica, acuminata, integerrima, nervofa, venofa, glabra, confiftentia. *Petiola partiales* breves, teretiufculi, glabri.

" *Racemi* axillares, compofiti, paniculati, ramis dichotomis patentibus, diffufis, multifloris.

" *Flores* parvi, albidi, polygami, mafculis et hermaphroditis in eodem racemo.

" Masc.

" Cal. 5-phyllus. *Foliola* conica, dentiformia, minuta.

" Cor. *Petala* 5 fub receptaculo inter dentes calycis inferta, oblonga, adfcendentia.

" Stam. *Filamenta* 5 a latere receptaculi exferta, fubulata, adfcendentia, petalis longiora, villofa. *Antheræ* fubglobofæ, bivalves.

" Pist. rudimentum.

" Hermaphrod.

" Cal. et Cor. ut in mare.

" *Filamenta* 5 breviora. *Antheræ* fertiles.

" Pist.

figures of the leaves and fructification, for which we muſt refer our readers to the work itſelf.

" Arbor excelſa ſæpe centum pedes alta.

" *Caudex* ſpectabilis, erectus, glaber. *Cortex*

" cinereus in Epidermide, interne albido fla-

" veſcens, tenax et ex fibris lentis confectus.

" *Ramuli* alterni teretes.

" *Folia* ſub-alterna. *Foliola* 5—10 jugata

" impari-pinnata, oppoſita, oblonga, obtuſe-

" acumi-

" Pist. *Germina* 3. contigua, receptaculo tumido inſi-

" dentia, globoſa, glaberrima. *Stylus* ſtaminibus longior,

" 3queter, 3-fidus. *Stigmata* ſimplicia.

" Per. *Drupæ* tres, globoſæ, uniloculares, bivalves, re-

" ceptaculo ampliato, hæmiſphærico inſidentes, diſtantes,

" magnitudine piſi majoris.

" Sem. *Nuces* ſolitariæ, globoſæ, glabræ, nauco fra-

" gili."

From this deſcription, Dr. Swartz obſerves, we may perceive the near affinity of this tree to the genus of Quaſſia. It approaches, he thinks, nearer to the Q. Simarouba than to the Q. Amara; but differs from both in being without the *ſquamulæ nectarii*, which are placed under the germen on the baſis of the filaments, and in having in general five, and ſometimes, though rarely, only four ſtamina inſtead of ten. Theſe two characteriſtic differences, however, are not, in his opinion, ſufficient to conſtitute a diſtinct genus.

" acuminata, glabra, integerrima, venofa, bre-

" viter petiolata. *Petiolus communis* fubtus nu-

" dus. *Stipulæ* laterales parvæ, lanceolatæ,

" erectæ, deciduæ.

" *Inflorefcentia* cymofa. *Pedunculi* folitarii,

" teretes, plerumque nudi, in plurimos ramu-

" los divifi.

" Flos Masculus.

" *Cal. Perianthium*, infernum, minimum, ex

" fquamulis quatuor compofitum. *Foliolis*

" ovatis perfiftentibus.

" *Cor. Petala* 4, oblonga, obtufa, æqualia, feffi-

" lia, fuberecta. *Nectarium* ex fquamis 4 ovatis,

" villofis, bafi filamentorum interiori infertis.

Dr. Swartz agrees with Mr. Lindfay in his account of the intenfely bitter tafte poffeffed by every part of the tree; and obferves that the negroes have recourfe to an infufion of it in rum in fome affections of the ftomach. He likewife mentions its ufe as an anthelmintic. His figure of the plant agrees with that given by Mr. Lindfay.—*See* Kongl. Vetenfkaps Academien nya Handlingar, Tom. 9, p. 302. 8vo. Stockholm, 1788. Editor.

" *Stam.*

" *Stam. filamenta* 4, 5, 6, filiformia, fube-

" recta, æqualia, corolla longiora, receptaculo

" inferta. *Antheræ* fimplices erectæ.

" Flos Hermaphroditus *in diverfa Arbore.*

" *Cal.* et *Cor.* ut in mare.

" *Stam.* ut in mare, fed filamenta corollam
" vix fuperant.

" *Pift.* Receptaculum carnofum, orbicula-
" tum, elevatum, germine latius. *Germen* fub-
" ovatum, ex duobus, tribus, raro quatuor
" compofitum, leviter coherentibus. Styli
" craffiufculi, erecti. Stigmata 2, 3, 4, fim-
" plicia, declinata.

" *Per.* Drupæ 2, 3, 4, globofæ, laterales,

" diftantes, nigerrimæ, nitentes, receptaculo

" infertæ.

" *Sem.* Solitaria globofa, unilocularia, nauco
" fragili tecta."

Mr. Lindfay next gives an account of the Cinchona Brachycarpa, a new fpecies of Cinchona growing in Jamaica. This tree was firft difcovered by him in November, 1784, on the north-eaſt ſide of the hill that overlooks the works of Mountain Spring eſtate, in the pariſh of Weſtmoreland, and afterwards on ſome of the mountains near the Moreland eſtates in the ſame pariſh. As it has hitherto been unknown to naturaliſts, he has given the following botanical account of it, which in the work itſelf is illuſtrated by an engraving.

" Pentandria Monogynia.

" *Cal.* Perianthium monophyllum, ſuperum,

" campanulatum, parvum, 5 dentatum, per-

" ſiſtens, dentibus acutis, erectis.

" *Cor.* Monopetala, infundibuliformis. *Tu-*

" *bus* cylindraceus longiſſimus. *Laciniis*, an-

" guſto oblongis, patente revolutis.

" *Stam.* Filamenta 5, interdum ſex, fili-

" formia, tubo longiora, in fauce tubi inſerta.

" *Antheræ* lineares erectæ.

" *Piſt.* Germen ovatum, inferum. Stylus

" filiformis longitudine ſtaminum. Stigma

" craſſiuſculum ovatum ſimplex.

" *Per.* Capſula oblongo ovata magna, calyce

" coronata, bipartibilis, dehiſcens in duas

" partes

" partes interius dehiſcentes, diſſepimento pa-

" rallelo.

" Semina plurima, parva, compreſſa, mar-

" ginata.

" Arbor erecta 20 pedes alta, ramis patenti-

" bus. Cortex fuſco-cinereus, ſapore primo

" dulci, mox amareſcente.

" Folia oppoſita, oblongo-ovata, integerri-

" ma, glabra, ſubtus venoſa, petiolata. Pe-

" tioli breves, ſupra ſulcati. *Stipulæ* laterales,

" ovato-lanceolatæ, integræ, caulem arcte am-

" plexantes.

" *Infloreſcentia* paniculato-corymboſa, termi-
" nalis. *Pedunculus* plerumque brachiato-tri-
" ternatus, teres, nudus. *Corolla* glabra, pal-
" lide rubra vel carnea, tres circiter pollices
" longa."

Mr. Lindſay has met with this tree only in three places; in the inland, woody, and mountainous parts of Weſtmoreland and Hanover pariſhes. The talleſt he has ever ſeen was about thirty feet high, and 7 or 8 inches in diameter. The branches, we are told, are few and ſpreading. The leaves ſtand in pairs, are ſmooth and ſhining, and are very like thoſe of the *Portlandia grandiflora*. The flowers grow in pretty large cluſters, on the extremities of the branches; and have nearly the beauty and appearance of the common honey fuckle, but are rather larger.

The feed-pod, he obferves, is larger than in any other plant of this genus; is oval, adorned with a calyx of a firm confiftence, fomewhat ftriated, and black-coloured; and when ripe, fplits in two, and difcharges a number of fmall, flat, brown feeds, with a membrane nearly round the edges.

The trunk and branches are defcribed as being of a brownifh gray colour, with a few fuperficial furrows, and crofs cracks like the Peruvian bark. The bark of the trunk, we are told, is pretty thick; and when wounded, exudes a fmall quantity of a milky juice. The bark, when dried, is of a purplifh brown colour on the infide. It is fibrous, and more difficult to pulverife than the Peruvian bark. The powder is of a purplifh gray colour, and taftes fweet, then bitter and aftringent.

This fpecies, our author thinks, might be ufed as a fubftitute to the Peruvian bark; but unfortunately the tree is fcarce and fmall, and enough of it cannot be had, at leaft in thofe parts of Jamaica in which he has found it growing. This lofs, he obferves, may be compenfated penfated by the abundance of the Cinchona Caribæa *feu* Jamaicenfis, defcribed by Dr. Wright in the 67th vol. of Phil. Tranf. and which, he is affured, has been found to anfwer all the purpofes of the Cinchona Officinalis.

He does not pretend to hold up this new bark as fuperior, or even equal to the Peruvian. He has given it, however, in the flighter cafes of intermitting and remitting fevers, with good effect; and in a few inftances, it produced a cure, where the patients had taken the common and red barks to no purpofe.

To perfons afflicted with intermittents, he gives of the powder from twelve grains to thirty every hour, or every two hours in the abfence of fever. By this means, a ftop has been put to the fever, and the patients have recovered. He has alfo adminiftered this new bark in dyfpepfia, both in powder and infufion, and found that it fat eafy on the ftomach, and promoted appetite. He had fhewn this fpecies of Cinchona to Dr. Wright, before he left Jamaica, and gave him a little of the bark. The Doctor gave it in powder to a patient, but found it emetic, which could only happen, our author thinks, from fome peculiarity of conconftitution *. In a letter to Mr. Lindfay, he intimates, that probably the fame thing would happen, with the bark of every other tree of this genus, if given before it is completely dried.

Mr. Lindfay's paper clofes with fome few remarks on the red Peruvian bark.

This fubftance, when genuine, and given brifkly in pretty large dofes, will, he obferves, in particular cafes, occafion a degree of anxiety, depreffion, giddinefs, and faintnefs, that are alarming to the patient and his friends, and perhaps, if not timely attended to, might be of ferious confequence. This, he adds, only happens in certain conftitutions, and in weakly habits, or thofe rendered fo by difeafe.

This effect of the red bark, fo far as he knows, has not been taken notice of by any writer, and when it occurs in private practice, is either not attended to, or imputed to fome other caufe. Mr. Lindfay gives the following extract of a letter from James Graham, Efq. a refpectable inhabitant of Jamaica, which, he thinks, places this circumftance in a ftrong light.

* See London Medical Journal, Vol. VIII. page 241.

Mr. Graham, after having been afflicted with a fever and ague for ſeveral months, at length took the red bark in doſes of thirty grains each. "On taking the firſt," ſays he, "I inſtantly perceived an unuſual pungency "on my tongue. After the fifth, I felt an "anxiety about my breaſt with faintiſhneſs; "and had hardly done ſwallowing the ſixth, "when I was ſeized with giddineſs, an uni- "verſal tremor, and a profuſe cold ſweat. A "little wine, which was given me in this ſitua- "tion, relieved me conſiderably. In about "an hour, all the alarming ſymptoms diſap- "peared, but I remained weak and languid. "From that day, however, the fever left me, "and did not return till ſeveral months after, "when it was brought on by a cold, and was "removed by the bark adminiſtered in the "ſame manner, and attended nearly by the "ſame ſymptoms as before."

